# Multi-omics integration identifies a gut-microbiota-associated plasma metabolite signature for alzheimer disease diagnosis and metabolic pathway targeting

**DOI:** 10.3389/fphar.2026.1933454

**Published:** 2026-08-19

**Authors:** Jiao Chen, Xuhui Chen, Weiwei Yu, Ting Chen, Zhigang Liu, Jun Hu

**Affiliations:** 1 Department of Neurology, Peking University Shenzhen Hospital, Shenzhen, China; 2 Department of Neurology, Peking University First Hospital, Beijing, China; 3 Department of Neurology, Shenzhen Second People’s Hospital, Shenzhen, China; 4 Huadong Hospital, Medicine-Agriculture Interdisciplinary Center, Institute of Nutrition, Fudan University, Shanghai, China; 5 Shenzhen Key Laboratory for Neuronal Structural Biology, Shenzhen Peking University-The Hong Kong University of Science and Technology Medical Center, Shenzhen, China

**Keywords:** alzheimer’s disease, biomarker, gut microbiota, gut–brain axis, metabolic pathway modulation, metabolomics, mitochondrial dysfunction, tricarboxylic acid cycle

## Abstract

**Background:**

Alzheimer’s disease (AD) is the leading cause of dementia, with early diagnosis remaining constrained by the invasiveness and limited accessibility of current biomarkers. The gut–brain axis has emerged as a critical communication network linking intestinal homeostasis with central nervous system function, yet the diagnostic utility of gut-microbiota-associated circulating metabolites in AD remains largely unexplored.

**Methods:**

In this case-control study, 85 participants (37 AD patients and 48 cognitively normal controls) were enrolled. Targeted plasma metabolomics using four complementary UPLC-MS/MS assays covering 375 endogenous metabolites was integrated with fecal metagenomic sequencing in 72 participants with paired samples. Differential metabolites were screened using the Mann–Whitney U test, orthogonal partial least squares discriminant analysis, and fold-change thresholds. Gut-microbiota-associated plasma metabolites were defined through Spearman correlation with linear discriminant analysis effect size-identified differential bacterial species. A diagnostic panel was constructed using least absolute shrinkage and selection operator logistic regression with stability selection, and its performance was evaluated by support vector machine with repeated cross-validation, with age and sex evaluated as covariates.

**Methods:**

We identified 27 differential plasma metabolites enriched in tricarboxylic acid cycle and amino acid metabolic pathways. Correlation analysis identified 10 gut-microbiota-associated plasma metabolites significantly associated with gut microbial alterations. A three-metabolite panel (citric acid, 3-hydroxyoctanoic acid, and glyoxylic acid) achieved a cross-validated area under the curve of 0.865, with a modest sensitivity of 62.2% and specificity of 100.0%, positioning it as a confirmatory auxiliary tool rather than a broad screening instrument. The panel score correlated negatively with Mini-Mental State Examination scores (r = −0.544, P < 0.001), and integration with clinical cognitive assessment yielded an area under the curve of 0.978.

**Conclusion:**

These findings potentially advance the translational understanding of the gut–brain metabolic axis in neurodegeneration and support the exploratory clinical utility of microbiota-linked peripheral metabolic signatures as non-invasive auxiliary diagnostic tools for AD, awaiting external validation in larger, multi-center cohorts.

## 1 Introduction

As the predominant cause of dementia, Alzheimer’s disease (AD) presents a pressing challenge to public health worldwide. The clinical features of AD include progressive memory loss, cognitive decline, and loss of functional independence. Pathologically, AD is characterized by the accumulation of amyloid-β (Aβ) plaques and tau neurofibrillary tangles, both of which are directly associated with synaptic damage and neurodegeneration (Bloom, 2014). Despite decades of research, the etiology and pathogenesis of AD remain incompletely understood, while early diagnosis is widely recognized as an effective means to improve survival rates in AD patients (Kastelan et al., 2024; Zhang et al., 2021b; Jack et al., 2024). Currently, the detection of Aβ and tau levels in cerebrospinal fluid (CSF), positron emission tomography (PET) imaging using Aβ and tau ligands, and magnetic resonance imaging (MRI) assessment of brain atrophy are established tools supporting the *in vivo* diagnosis of AD (Aramadaka et al., 2023; Tanaka et al., 2025; Patil et al., 2025; Quinn and Gray, 2024; Shaw et al., 2018). However, the widespread clinical application of these methods is still limited by factors such as invasive procedures (e.g., lumbar puncture), high costs, limited accessibility, and radiation exposure (Yuan and Lee, 2022). In recent years, plasma-based phosphorylated tau proteins (e.g., pTau181, pTau217) and neurofilament light chain (NFL) assays have emerged as promising minimally invasive alternatives (Thijssen et al., 2022; Benedet et al., 2022; Chatterjee et al., 2023; Groot et al., 2022; Ashton et al., 2024). A growing body of evidence suggests that AD is a multifactorial disease, in which peripheral metabolic dysregulation, mitochondrial dysfunction, and systemic inflammation play important roles in disease onset and progression (Walker et al., 2023; Peng et al., 2020; Liao et al., 2024). However, existing assays primarily reflect the downstream pathological consequences of Aβ and tau accumulation, failing to capture the broader concomitant systemic metabolic disturbances. Therefore, it is urgent to discover minimally invasive biomarkers that can reflect the systemic pathological process of AD, and integrate them into the existing clinical diagnostic system to improve the accuracy of AD diagnosis.

Although AD is considered a brain disorder, mounting evidence indicates that non-brain factors—including genetic polymorphisms, metabolic dysregulation, immune dysfunction, and environmental influences—can also contribute to disease initiation and exacerbation, highlighting the necessity of integrative research approaches that extend beyond the central nervous system (CNS) (Bellenguez et al., 2022; Vogt et al., 2017; Heneka et al., 2015). The role of gut microbiota in human disease pathogenesis has garnered growing research interest. Its composition and functional capacity are dynamically shaped by multiple host-intrinsic and extrinsic factors, notably dietary patterns, which collectively modulate microbial-derived metabolites and host-microbe interactions (Zhao Y. et al., 2025). Over the past decade, the gut-brain axis (GBA) has emerged as a critical communication network linking intestinal homeostasis with CNS function, and its dysfunction has been implicated in the pathogenesis of various neurodegenerative diseases, including AD (Morais et al., 2021; Kapoor et al., 2024; Jamerlan et al., 2025). In AD patients, the composition of gut microbiota may undergo significant alterations, manifesting as reduced microbial diversity, decreased beneficial commensal bacteria, and overgrowth of pro-inflammatory taxa, which are closely associated with cognitive decline, increased Aβ deposition, elevated tau phosphorylation levels, and neuroinflammation (Vogt et al., 2017; Seo et al., 2024; Carranza-Naval et al., 2021; Wasen et al., 2022; Asadifard et al., 2024). These compositional changes are accompanied by alterations in microbial metabolic functions, leading to disturbances in the circulating metabolome (Luo et al., 2024; Zhao H. et al., 2025).

AD is accompanied by global alterations in metabolic status, affecting both the central nervous system microenvironment and the systemic milieu. Metabolomics has been increasingly employed in AD research to identify metabolic signatures and characterize the biological changes that occur during disease initiation and progression (Lista et al., 2023). Consequently, metabolomics can be utilized to discover novel AD biomarkers and may inform diagnostic, prognostic, and precision therapeutic strategies. Multiple studies have demonstrated that metabolites produced by intestinal bacteria can enter the bloodstream and exert regulatory functions in distal organs (Tang et al., 2019; Zhang et al., 2021a). Metabolomics, defined as the comprehensive profiling of small-molecule metabolites, provides a powerful systems-level approach for characterizing the effects of gut microbiota dysbiosis on host physiology and its downstream consequences. In recent years, plasma metabolites closely associated with AD have garnered substantial attention for the development of diagnostic biomarkers (Zhang et al., 2024). However, most existing studies have focused exclusively on either gut microbiota or the metabolome in isolation, and the impact of AD-related microbial changes on blood metabolites, as well as the clinical significance of these changes, remains unclear.

In the present study, we performed plasma targeted metabolomics analysis using four complementary ultra-performance liquid chromatography-tandem mass spectrometry (UPLC-MS/MS) platforms and combined this with metagenomic sequencing of paired fecal samples to screen for significantly altered gut microbiota-associated plasma metabolites in AD patients. Based on these metabolites, we constructed a model comprising three gut microbiome-associated plasma metabolites (the GMPM panel), explored the clinical significance of this panel, and evaluated its diagnostic value when integrated with clinical variables. We anticipate that this GMPM model will provide an auxiliary diagnostic tool with clinical utility for AD and offer novel insights into the peripheral metabolic consequences of gut microbiota dysbiosis in neurodegenerative diseases.

## 2 Materials and methods

### 2.1 Study participants and grouping

**FIGURE 1:**
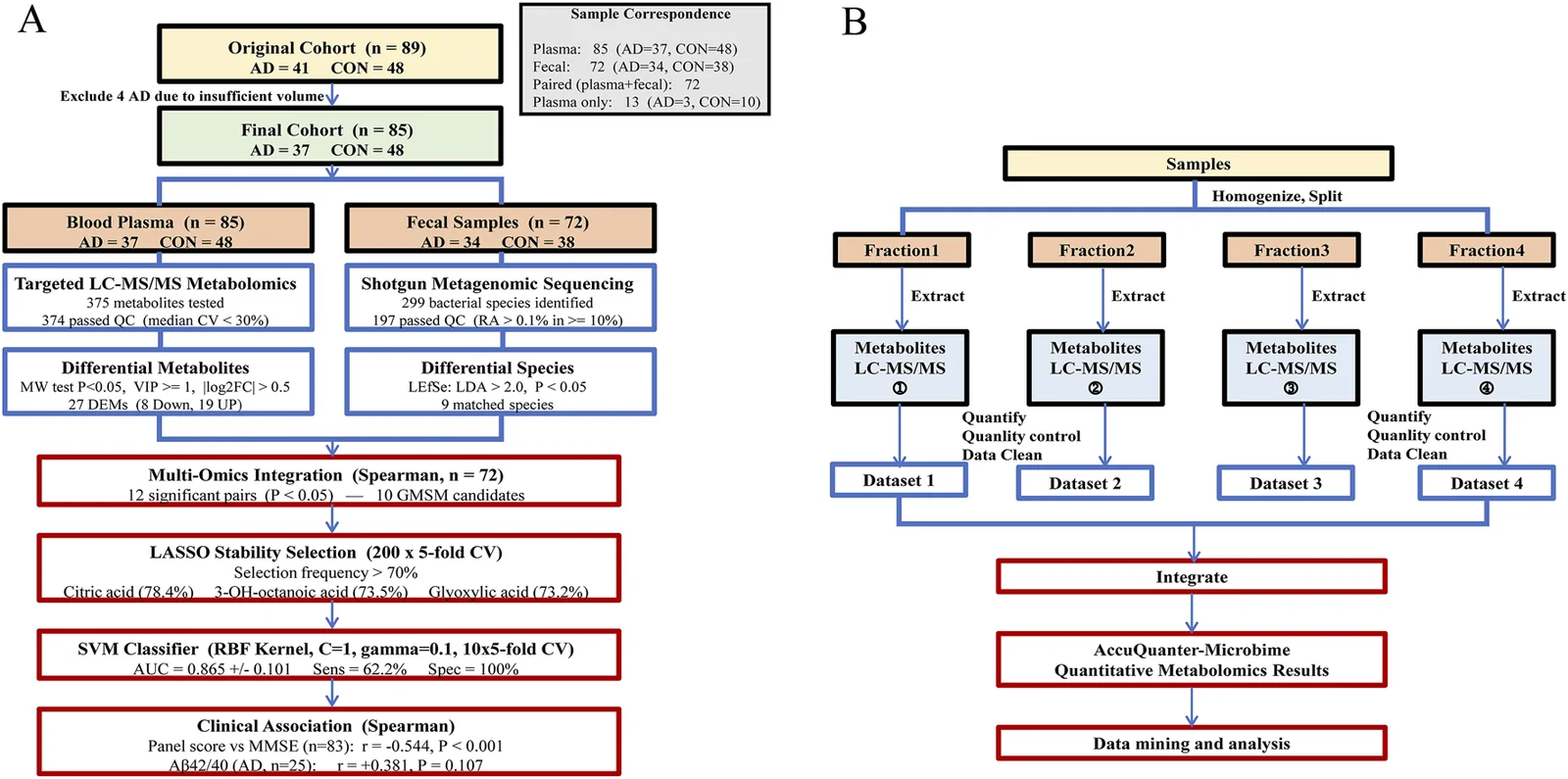
Study design and metabolomics profiling workflow. **(A)** Flowchart of participant recruitment, screening, and group allocation. A total of 89 participants were recruited, comprising 37 patients with Alzheimer’s disease (AD) and 48 cognitively normal controls. After excluding four participants due to insufficient sample volume, 85 participants were included in the plasma metabolomics analysis. Among these, 72 participants with paired fecal and plasma samples were included in the multi-omics integration analysis **(B)** Schematic overview of the targeted plasma metabolomics platform. Metabolites were quantified using the AccuQuanter-Microbiome panel on the I-Phenomene platform. The analytical pipeline comprised four complementary liquid chromatography–tandem mass spectrometry (LC-MS/MS) assays.

Participants were enrolled in a case-control framework at the Neurology Department of Peking University Shenzhen Hospital. No formal sample size calculation was performed for this exploratory study. The sample size was determined pragmatically based on the availability of participants meeting eligibility criteria during the recruitment period, and was considered adequate for a preliminary evaluation of a multi-omics diagnostic model, consistent with similar proof-of-concept studies in the field. A total of 89 consecutive participants were recruited, comprising 37 individuals with clinically diagnosed AD and 48 cognitively normal controls (Control). Four participants were excluded due to insufficient sample volume for metabolomic analysis, leaving a final cohort of 85 participants (37 AD patients and 48 controls) included in the plasma metabolomics analysis. Among these, 72 participants (34 AD patients and 38 controls) had paired fecal and plasma samples and were included in the multi-omics integration analysis (Figure 1A).

All experimental procedures adhered to the ethical principles outlined in the Declaration of Helsinki. The study protocol received approval from the Institutional Review Board (IRB) of Peking University Shenzhen Hospital (approval number: 2024–151; approval date: 30 October 2024), and written informed consent was secured from all participants before enrollment.

Eligibility was determined using predefined inclusion and exclusion criteria. For the AD cohort, participants were required to meet the 2018 NIA-AA criteria for mild-to-moderate cognitive impairment due to AD, with diagnostic confirmation via positive blood-based biomarkers, cerebrospinal fluid markers, or PET-CT imaging. Age eligibility was set at ≥50 years, with no restriction on sex. The control group comprised cognitively intact individuals verified by the Mini-Mental State Examination (MMSE). AD severity was staged according to MMSE scores: 21–24 (mild), 10–20 (moderate), and 0–9 (severe).

Individuals were excluded if they had a documented history of inflammatory bowel disease or other serious medical conditions; comorbid neurological disorders (e.g., Parkinson’s disease, stroke); current use of medications known to alter gastrointestinal function; or unwillingness to provide informed consent. Baseline demographic and clinical characteristics, including sex and age at onset, were systematically captured using standardized case report forms to ensure comprehensive clinical profiling. Di-agnostic classification was finalized prior to the initiation of metabolomic analyses.

### 2.2 Sample collection and storage

#### 2.2.1 Plasma sample collection

Five milliliters of whole blood were collected from each participant via venipuncture and transferred into EDTA vacutainer tubes (BD Diagnostics). Samples were im-mediately centrifuged at 4 °C and 1,500 × g for 15 min. The resulting supernatant plasma was subsequently aliquoted into 1.5 mL polypropylene tubes and stored at −80 °C until metabolomic analysis.

To ensure temporal alignment between biological and clinical data, biospecimen collection was performed concomitant with neuropsychological and diagnostic assessments. Participants were required to observe an overnight fast (≥12 h) prior to phlebotomy to mitigate postprandial fluctuations in circulating metabolites. Blood draws were performed by certified phlebotomists under aseptic conditions. Cognitive evaluations and neuroimaging followed standardized clinical protocols. No adverse events attributable to blood sampling or diagnostic procedures were documented throughout the study.

#### 2.2.2 Fecal sample collection

Fecal samples were collected from participants who had also provided fasting plasma samples for metabolomic analysis. Participants were instructed to defecate after placing a disposable medical fecal collection paper on the toilet seat to prevent contamination from urine and toilet surfaces. Approximately 5–10 g of fresh fecal material was collected from the central portion of the stool specimen using a sterile collection spoon and immediately transferred into a sterile fecal collection container. Participants were explicitly instructed to avoid collecting surface portions or regions potentially exposed to urine or toilet water. Immediately following collection, fecal containers were placed in a portable refrigerated transport box (0 °C–4 °C) and delivered to the laboratory for processing within 1 h. The cold chain was maintained throughout the entire transportation period. Upon arrival at the laboratory, each fecal sample was processed promptly under sterile conditions. Aliquots of approximately 200–500 mg were dispensed into pre-labeled sterile cryovials (1.5 mL or 2.0 mL) using sterile spatulas. Mul-tiple aliquots were prepared from each sample to allow for replicate analyses and future use. All aliquoted samples were immediately transferred to an ultra-low temperature freezer at −80 °C and stored longitudinally until subsequent DNA extraction and metagenomic sequencing.

### 2.3 Plasma metabolomics profiling

Plasma metabolites were quantified using the AccuQuanter-Microbiome panel (Biotree Biomedical Technology Co., Ltd., Shanghai, China) on the IPhenomene platform (Biotree Biomedical Technology Co., Ltd). The analytical pipeline comprised four complementary LC-MS/MS assays performed on an AB SCIEX 5500+ triple quadrupole mass spectrometer (AB SCIEX, Framingham, MA, United States) coupled with a Shimadzu LC-20ADXR UHPLC system (Shimadzu Corporation, Kyoto, Japan). Metabolites were detected using scheduled multiple reaction monitoring (MRM) with stable isotope-labeled internal standards for absolute quantification. The four assay modules collectively targeted 375 endogenous metabolites across major metabolic classes: (i) 133 polar metabolites (amino acids, nucleotides, and water-soluble vitamins) via hydrophilic interaction chromatography; (ii) 111 lipid-related metabolites (bile acids and fatty acids) via reversed-phase chromatography; (iii) 86 carboxylic acid- and aldehyde-containing metabolites (short-chain fatty acids and tricarboxylic acid cycle intermediates) after 3-nitrophenylhydrazine derivatization; and (iv) 108 additional metabolites encompassing polyamines and aromatic amino acid catabolites (Lv et al., 2022; Han et al., 2015) (Figure 1B).

Raw concentration data (nmol/L) were preprocessed in Python 3.11 (Python Software Foundation, Wilmington, DE, United States) using NumPy 1.26.4 (Van Der Walt et al., 2011), pandas 2.1.4 (McKinney WJPfhp, 2011), and scikit-learn 1.3.2 (Pedregosa et al., 2011). Metabolites with >20% missing values across samples were removed; remaining missing values were imputed by k-nearest neighbors (KNN; sklearn. impute.KNNImputer, k = 5). Data were then subjected to probabilistic quotient normalization (Dieterle et al., 2006), followed by log_2_ transformation (with a +1 × 10^−6^ offset to avoid log of zero). Four pooled quality control (QC) samples were inserted into the injection sequence to monitor system stability. Each pooled QC sample was prepared by mixing equal volumes of plasma from 30 randomly selected participants. The quantitative reliability of each metabolite was assessed by the coefficient of variation (CV = σ/μ) across the four QC replicates. Only metabolites with a median CV below 30% were retained for further analysis. QC reproducibility was further evaluated by pairwise Pearson correlation among QC samples.

Differential metabolites between the AD and control groups were screened using three complementary criteria applied in parallel: (i) Mann–Whitney U test (scipy.stats.mannwhitneyu, two-sided) P < 0.05 (MacFarland and Yates, 2016), (ii) variable importance in projection (VIP) score ≥1.0 from Orthogonal Partial Least Squares Discriminant Analysis (OPLS-DA) (Farrés et al., 2015; Worley and Powers, 2016) conducted in SIMCA 14.1 (Sartorius Stedim Data Analytics AB, Umeå, Sweden) (Robotti and Marengo, 2016), (iii) absolute log_2_ fold change (|log_2_ FC|) > 0.5, corresponding to a >1.41-fold difference in raw concentration.

Principal component analysis (PCA) was performed on the Z-score-normalized abundances of the differential metabolites (sklearn.decomposition.PCA, n_components = 2, with prior sklearn. preprocessing.StandardScaler). A heatmap was constructed in R 4.3.3 (Chan, 2018) using pheatmap 1.0.12 (Kolde and Kolde, 2015), with hierarchical clustering of metabolite rows via the Ward. D2 algorithm (Murtagh et al., 2011) on Euclidean distances of Z-score-normalized intensities, while sample columns remained unclustered. Figures were generated using ggplot2 3.5.1 (Wickham HJWircs, 2011), ggrepel 0.9.5 (Slowikowski et al., 2024), and patchwork 1.2.0 (Pedersen TLJRphCR-poppC, 2019). Kyoto Encyclopedia of Genes and Genomes (KEGG) pathway enrichment analysis was implemented using clusterProfiler 4.10.0 (Wu et al., 2021) via over-representation analysis with the hypergeometric test (Falcon and Gentleman, 2008), using the full set of 375 assayed metabolites as the reference background; the Benjamini–Hochberg false discovery rate (FDR) was applied for multiple-testing correction. The top 10 enriched pathways ranked by raw P-value are presented in the bubble plot.

Participants with equivocal clinical diagnoses or insufficient bio-sample quality for reliable metabolomic quantification were excluded prior to analysis. Four participants were excluded due to insufficient plasma volume, as described in Section 2.1. For metabolites with >20% missing values across samples, the metabolite was excluded from downstream analysis; remaining missing values were imputed using k-nearest neighbor imputation (k = 5). No indeterminate diagnostic classifications were retained in the final analytic cohort.

### 2.4 Fecal metagenomics

Fecal samples were subjected to whole-genome shotgun metagenomic sequencing (Quince et al., 2017). Taxonomic profiling was performed against a standardized reference database constructed using RESCRIPt (Robeson et al., 2021), identifying a total of 299 bacterial species. Species with relative abundance >0.1% in ≥10% of samples were retained for downstream analysis. Alpha diversity (Shannon and Simpson indices) and beta diversity (Bray-Curtis dissimilarity with PCoA) were compared between groups (Socolar et al., 2016). Principal Coordinates Analysis (PCoA) based on Bray–Curtis dissimilarity matrices was performed by eigendecomposition using SciPy 1.17.1 (Virtanen et al., 2020). Linear Discriminant Analysis (LDA) Effect Size (LEfSe) analysis was conducted via the Galaxy web platform (Huttenhower Lab, Harvard T.H. Chan School of Public Health, Boston, MA, United States) with thresholds of LDA score >2.0 and P < 0.05 to identify differentially abundant species (Chang et al., 2022).

### 2.5 Multi-omics integration

Spearman’s rank correlation analysis was performed using SciPy 1.17.1 (scipy.stats.spearmanr, two-sided) to evaluate the associations between differential plasma metabolites and LEfSe-identified differential bacterial species across all participants with paired fecal and blood samples. Metabolites exhibiting a significant correlation with at least one bacterial species (P < 0.05) were defined as GMPMs. The full-cohort analysis was adopted as the primary analytical strategy to avoid confound-ing bias inherent in association analyses restricted solely to the disease subgroup.

### 2.6 Diagnostic panel construction

Least absolute shrinkage and selection operator (LASSO) logistic regression (Zou, 2006) with stability selection (scikit-learn, sklearn. linear_model.LogisticRegression, 200 repeats × 5-fold cross-validation, C = 0.1) was applied to the GMPM candidates to identify the most robustly selected metabolites. Metabolites with a selection frequency >70% were retained to form the final diagnostic panel. A support vector machine (SVM) (Hearst et al., 1998) classifier with a radial basis function (RBF) kernel (scikit-learn, sklearn. svm.SVC, C = 1, γ = 0.1) was trained on the selected panel metabolites. Model performance was evaluated using repeated stratified 5-fold cross-validation (scikit-learn, sklearn. model_selection.RepeatedStratifiedKFold, 10 repeats), and the area under the receiver operating characteristic curve (AUC), sensitivity, and specificity are reported as mean ± standard deviation.

### 2.7 Clinical association

SVM-derived panel scores were correlated with cognitive function assessed by MMSE and CSF Aβ42/40 ratios using Spearman’s rank correlation. To evaluate the incremental diagnostic value of combining metabolomic and clinical variables, logistic regression models were constructed using scikit-learn (sklearn.linear_model.LogisticRegression, max_iter = 10,000): (i) a base model with the metabolite panel score alone, and (ii) an integrated model combining the panel score with age, sex, and MMSE. Receiver operating characteristic (ROC) curves were generated for both models, and their discriminative performance was compared (scikit-learn, sklearn. metrics.roc_curve).

## 3 Results

### 3.1 Demographic and disease characteristics

Demographic and clinical characteristics of the study participants are summarized in Table 1. A total of 85 participants were included in the analysis, comprising 37 AD patients and 48 cognitively normal controls. The mean age was comparable between the two groups (AD: 65.0 ± 11.3 years; controls: 62.3 ± 8.4 years; independent t-test, P = 0.211). However, a significant difference in sex distribution was observed, with a higher proportion of females in the AD group compared with the control group (67.6% [25/37] versus 39.6% [19/48]; chi-square test, P = 0.019). As expected, AD patients exhibited significantly lower MMSE scores than controls (median [IQR]: 19 [16–24] versus 30 [28–30]; Mann–Whitney U test, P < 0.001). Among the AD patients, disease severity was classified as mild in 13 (35.1%), moderate in 17 (45.9%), and severe in 7 (18.9%) individuals. CSF biomarkers (Aβ42/40 ratio and pTau) were available in a subset of AD patients and but not for cognitively normal controls. Given that lumbar puncture is an invasive procedure, and cognitively intact individuals had no clinical indication for CSF collection, ethical considerations precluded invasive sampling in the control group. Consequently, direct comparisons of CSF biomarkers between groups were not performed in this study.

**TABLE 1:** Demographic and clinical characteristics of study participants.

Characteristic		AD (n = 37)	Control (n = 48)
Sex, n (%)	Female	25 (67.6)	19 (39.6)
	Male	12 (32.4)	29 (60.4)
Age, years, mean ± SD		65.0 ± 11.3	62.3 ± 8.4
MMSE score, median (IQR)		19 (16–24)	30 (28–30)
CSF Aβ42/40 ratio, median (IQR)		0.0500 (0.0400–0.0500)	Not measured
CSF pTau, pg/mL, median (IQR)		101.4 (68.3–129.6)	Not measured
Disease stage, n (%)	Mild	13 (35.1)	—
	Moderate	17 (45.9)	—
	Severe	7 (18.9)	—

MMSE, Mini-Mental State Examination; CSF, cerebrospinal fluid; Aβ = amyloid-β; pTau, phosphorylated tau; IQR, interquartile range. Disease stage was assessed only in AD, patients: 0–9, severe; 10–20, moderate; 21–24, mild. Statistical tests: independent t-test for age; Mann–Whitney U test for MMSE; chi-square test for sex. CSF, biomarkers were measured only in a subset of AD, patients.

### 3.2 Plasma metabolomics analysis revealed the significantly altered metabolites in the bodies of AD patients

**FIGURE 2:**
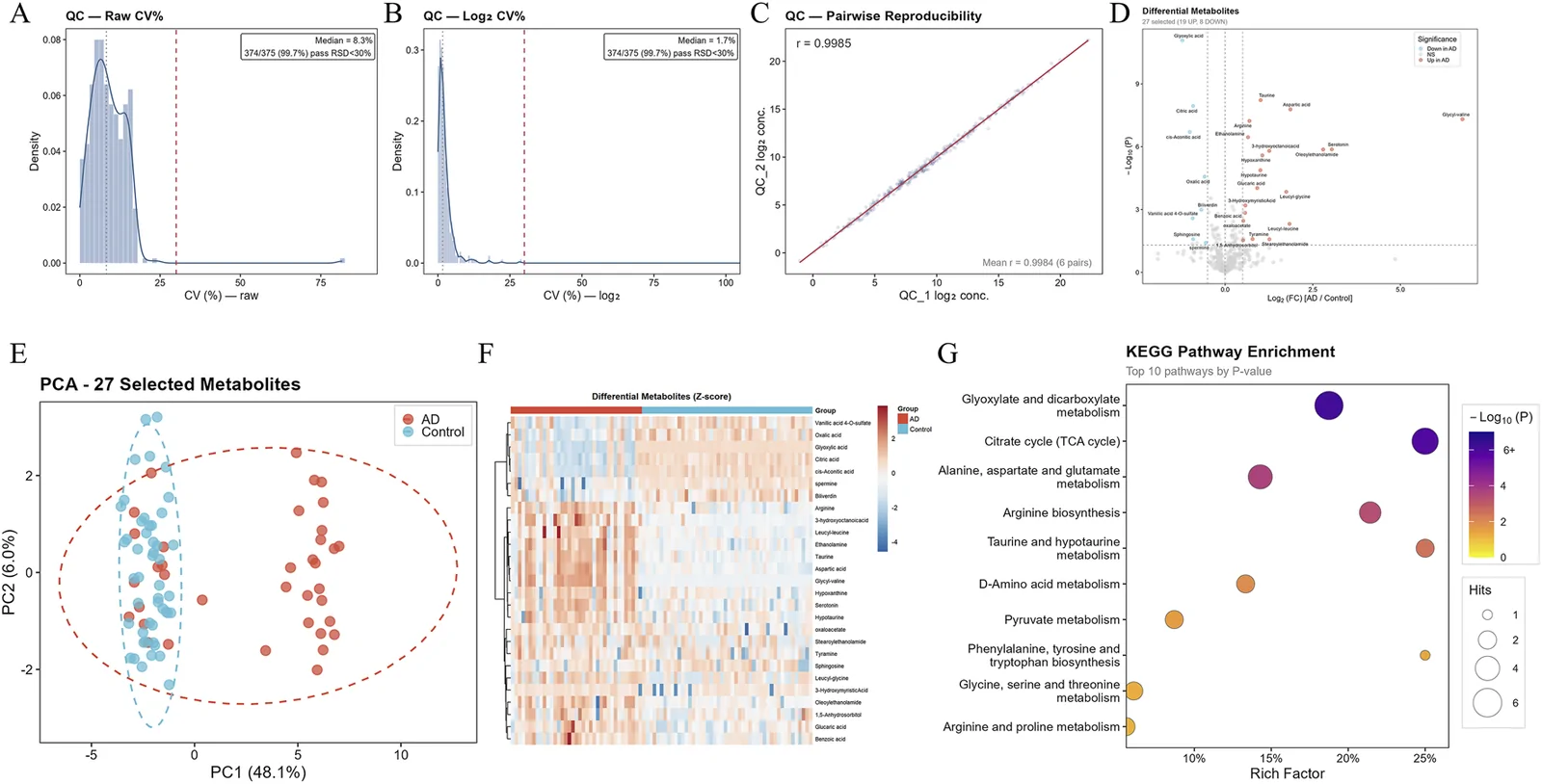
Quality control and differential analysis of plasma metabolomics. **(A)** Density distribution of raw coefficient of variation (CV%) for all detected metabolites. **(B)** Density distribution of log_2_-transformed CV% for all detected metabolites. **(C)** Pairwise reproducibility scatter plot of quality control (QC) samples. **(D)** Volcano plot of differential metabolites between AD patients and cognitively normal controls. **(E)** Principal component analysis (PCA) score plot based on the 27 differential metabolites. **(F)** Heatmap of the 27 differential metabolites. **(G)** KEGG pathway enrichment bubble plot of the 27 differential metabolites.

Rigorous technical QC was performed for the targeted LC-MS metabolomic dataset covering 375 endogenous plasma metabolites. The median coefficient of variation (CV) of raw metabolite concentrations was 8.25% (Figure 2A). After log_2_ transformation, the median CV decreased to 1.66% (Figure 2B). A total of 374 out of 375 detected metabolites (99.7%) passed the QC threshold of median CV < 30%, confirming the high analytical stability of the platform. The pairwise Pearson correlation coefficient among QC replicates reached r = 0.9985, demonstrating excellent technical re-producibility (Figure 2C).

Using three complementary statistical filtering criteria, we identified 27 differential metabolites between patients with AD and cognitively normal controls. Among these, 19 were elevated and 8 were decreased in the AD group (Figure 2D). PCA based on Z-score-normalized intensities of the 27 differential metabolites revealed clear group separation along PC1, which explained 48.1% of the total metabolic variation (Figure 2E). Metabolic heterogeneity among AD participants was greater than that among control subjects. Hierarchical clustering of standardized metabolite profiles revealed a distinct AD-associated metabolic signature (Figure 2F). The elevated metabolites in AD were predominantly amino acids and their derivatives, including glycyl-valine, serotonin, aspartic acid, leucyl-leucine, taurine, hypo-taurine, and oxalo-acetate. Conversely, the decreased metabolites were primarily organic acids linked to the tricarboxylic acid (TCA) cycle and related pathways, including glyoxylic acid, cis-aconitic acid, citric acid, and oxalic acid.

KEGG pathway over-representation enrichment analysis was performed to interpret the functional relevance of these altered metabolites (Figure 2G). Four pathways showed significant enrichment with FDR <0.05: glyoxylate and dicarboxylate metabolism, citrate cycle (TCA cycle), alanine, aspartate and glutamate metabolism, and argi-nine biosynthesis. These enriched pathways are centered on core energy metabolism and amino acid homeostasis, revealing a systemic metabolic reprogramming in AD. The concurrent depletion of both canonical TCA cycle intermediates (citric acid, cis-aconitic acid) and the glyoxylate/dicarboxylate metabolite glyoxylic acid, together with the elevation of oxaloacetate and multiple amino acids, suggests a complex disruption of mitochondrial carbon metabolism rather than a simple shift toward alternative pathways. This metabolic pattern is consistent with well-established AD neuro-pathological hallmarks, including mitochondrial dysfunction and impaired cerebral glucose metabolism. Notably, the pronounced depletion of glyoxylic acid relative to elevated oxaloacetate levels suggests dysregulation of glyoxylate/dicarboxylate metabolism under AD-related mitochondrial stress.

### 3.3 Gut metagenomics has revealed the changes in the gut microbiota of patients with AD

**FIGURE 3:**
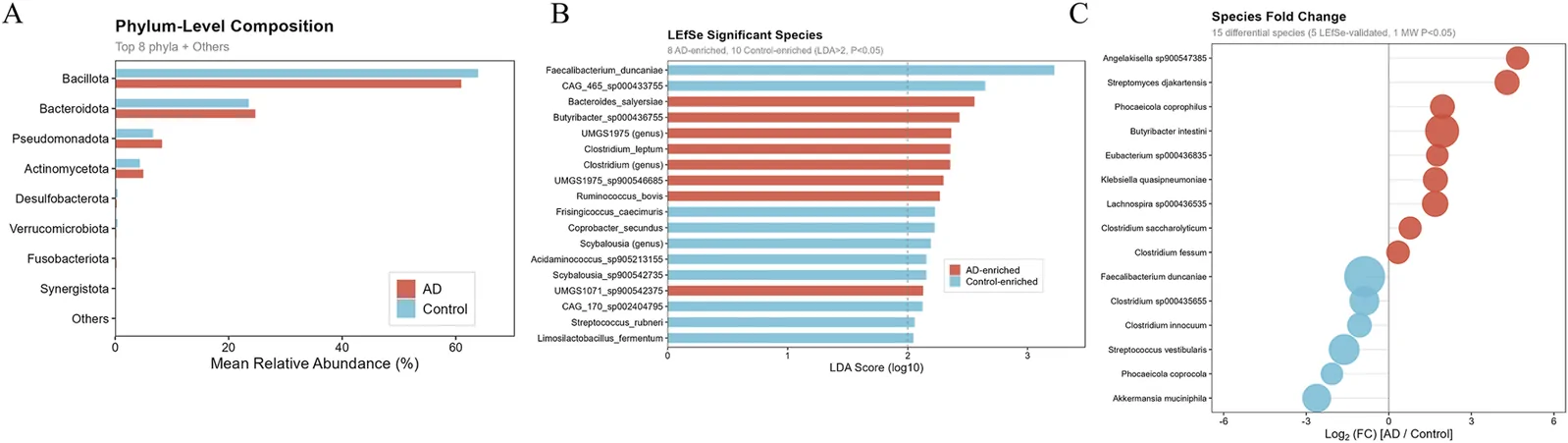
Gut metagenomic profiling and differential species analysis. **(A)** Phylum-level taxonomic composition showing the relative abundance of the 13 detected bacterial phyla in AD and control groups. **(B)** Linear discriminant analysis (LDA) effect size (LEfSe) bar plot of 18 differentially abundant species (LDA score >2.0, P < 0.05). Species enriched in the control group are shown on the left (negative LDA scores), and species enriched in the AD group are shown on the right (positive LDA scores). **(C)** Species-level fold-change analysis of 15 differential species between AD and control groups. Data are presented as log_2_ fold-change values, with the direction of change indicating enrichment (positive) or depletion (negative) in the AD group relative to controls. LEfSe, linear discriminant analysis effect size; LDA, linear discriminant analysis.

Taxonomic profiling of fecal metagenomic data was performed on paired fecal samples (34 AD patients and 38 cognitively normal controls). At the phylum level, the gut microbial community in both groups was dominated by *Bacillota* and *Bacteroidota*. *Bacillota* accounted for 61.00% of the total relative abundance in the AD group and 63.96% in the control group, while *Bacteroidota* represented 24.76% and 23.60%, respectively. No statistically significant differences were observed in the relative abundance of all 13 detected phyla between the two groups (all P > 0.05; Figure 3A).

Alpha diversity indices, including Shannon and Simpson, showed no significant differences between AD and control subjects. Beta diversity analysis based on Bray–Curtis dissimilarity revealed no clear separation according to disease status. These results suggest that microbial alterations associated with AD occur at a refined taxonomic level rather than at the level of the overall community structure.

Linear discriminant analysis (LDA) effect size (LEfSe) analysis identified 18 species as biomarkers with LDA scores >2.0 and P < 0.05. Among these, 8 species were enriched in the AD group and 10 species were enriched in the control group (Figure 3B). Notable species enriched in the control group included *Faecalibacterium duncaniae* and *Limosilactobacillus fermentum*, both of which were known as anti-inflammatory short-chain fatty acid (SCFA)-producing microorganisms. *Faecalibacterium duncaniae* exhibited the strongest discriminatory capacity, with an LDA score of 3.22. Conversely, several species enriched in the AD group, such as *Clostridium leptum* and *Ruminococcus bovis*, were associated with pro-inflammatory metabolism.

Species-level fold-change analysis identified 15 differential species (Figure 3C). *Faecalibacterium duncaniae* was significantly depleted in the AD group, with a log_2_ fold-change of −0.878 (Mann–Whitney U test, P = 0.036). However, after false discovery rate (FDR) correction, no species remained statistically significant. These findings indicate that the subtle magnitude of gut microbiota alterations in AD—compounded by substantial inter-individual variation—likely constrained statistical power in our cohort (n = 85). Nevertheless, the directional consistency of our findings (e.g., depletion of butyrate-producing taxa in AD) aligns with established literature (Sepulveda-Rivera et al., 2025), suggesting that these species represent biologically plausible, albeit modestly powered, candidates warranting validation in larger cohorts.

### 3.4 Comprehensive microbiome-metabolome analysis revealed the plasma metabolites related to the gut microbiota associated with AD

**FIGURE 4:**
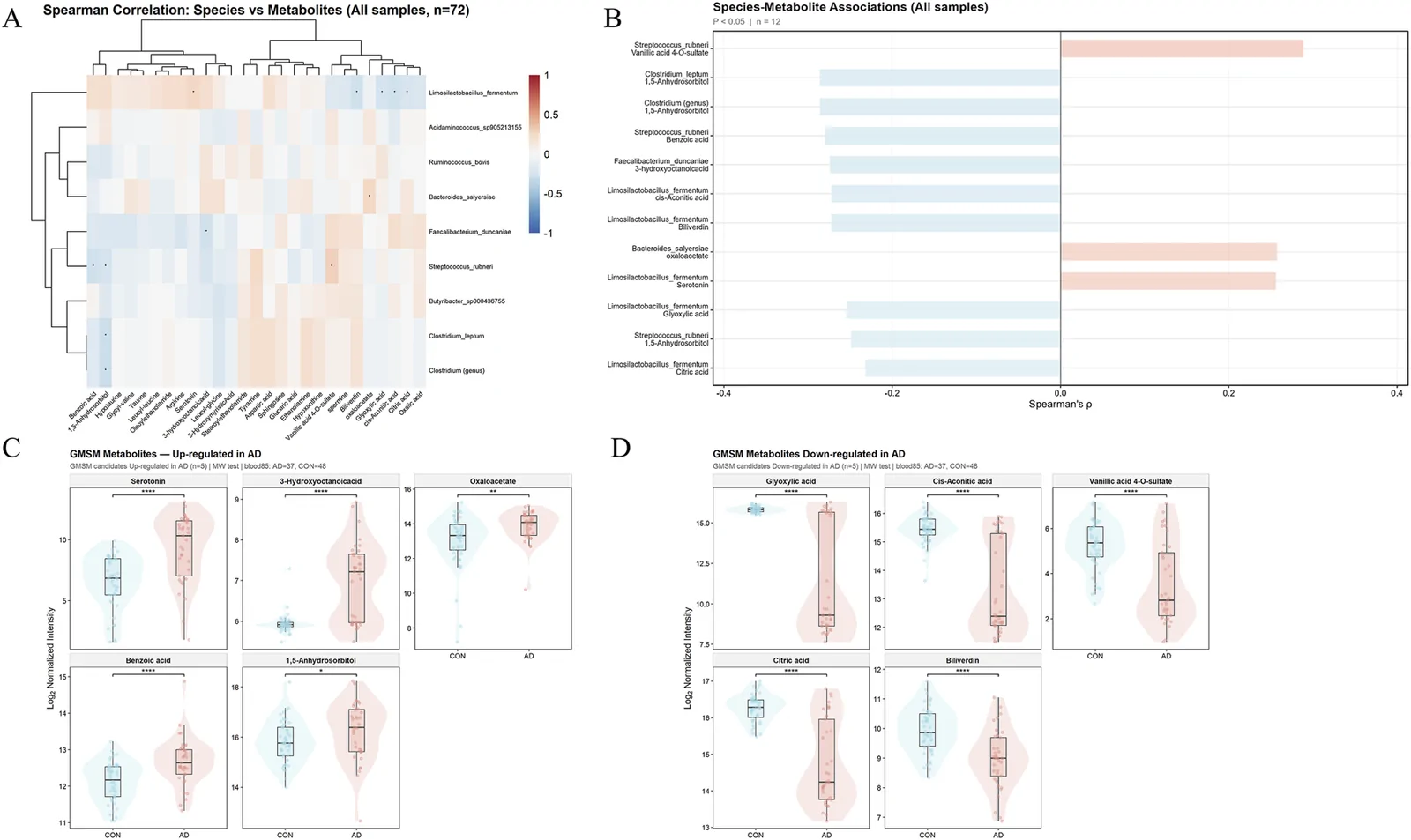
Multi-omic integration reveals gut microbiota–plasma metabolite associations. **(A)** Spearman correlation heatmap between differential bacterial species and differential plasma metabolites. **(B)** Bar chart of the 12 significant bacteria–metabolite pairs (P < 0.05). **(C,D)** Box plots showing the abundance patterns of the 10 Gut-microbiota-associated plasma metabolites (GMPMs) in AD versus control subjects: five metabolites significantly elevated in the AD group **(C)** and five significantly decreased **(D)**.

Spearman’s rank correlation analysis was performed to assess associations be-tween differential bacterial species and differential plasma metabolites among participants with paired stool and blood samples. The full correlation matrix was visualized as a heatmap (Figure 4A). Twelve significant bacteria–metabolite pairs (P < 0.05) were identified, and their correlation coefficients are presented in the bar chart (Figure 4B). After removing duplicate metabolites, 10 unique GMPMs were retained. These associations primarily involved commensal bacteria, including *Limosilactobacillus fermentum*, *Streptococcus rubneri*, and *F. duncaniae*, which were correlated with several organic acid metabolites, bile pigments, and neurotransmitters. These results indicate an interplay between the gut microbial community and circulating metabolites in AD patients.

The abundance patterns of the 10 GMPMs in AD versus control subjects are presented in Figures 4C,D. Among them, five metabolites were significantly elevated in the AD group: serotonin, oxaloacetate, 3-hydroxyoctanoic acid, benzoic acid, and 1,5-anhydrosorbitol. The remaining five were significantly decreased: glyoxylic acid, cis-aconitic acid, vanillic acid 4-O-sulfate, citric acid, and biliverdin. Notably, glyoxylic acid and cis-aconitic acid, both intermediates of the TCA cycle and glyoxylate/dicarboxylate metabolism, exhibited the most pronounced depletion in AD, while serotonin showed the greatest elevation. This bidirectional pattern aligns with the broader differential metabolomic profile and highlights a subset of microbiota-linked metabolites whose circulating levels are coordinately altered in AD.

### 3.5 Diagnostic metabolite panel construction and clinical association

**FIGURE 5:**
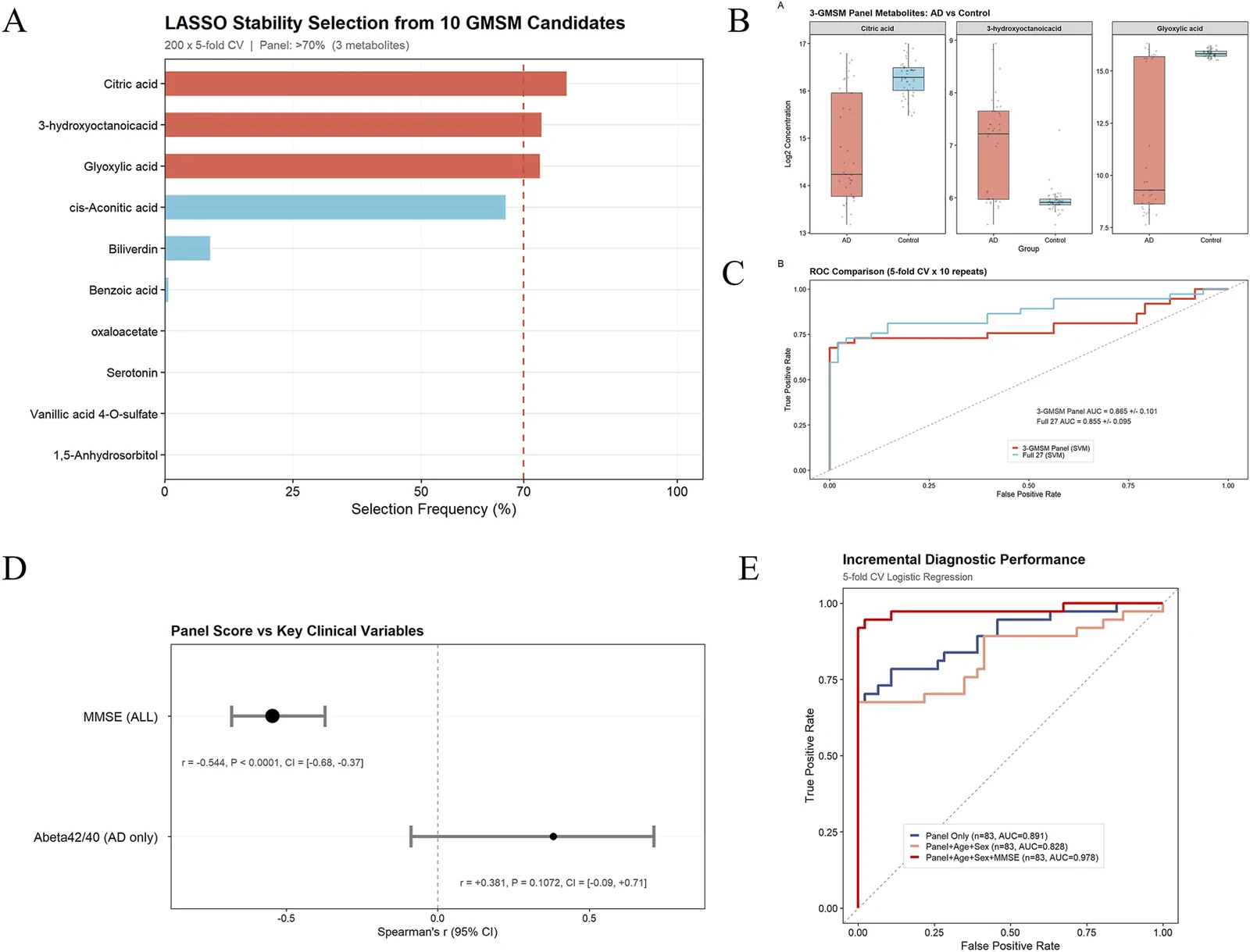
Diagnostic metabolite panel construction and clinical validation. **(A)** LASSO stability selection bar plot showing the selection frequencies of the three panel metabolites (citric acid, 3-hydroxyoctanoic acid, and glyoxylic acid) across 200 iterations of 5-fold cross-validation. **(B)** Box plots of the three selected panel metabolites comparing abundance levels between AD and control groups. **(C)** ROC curve comparison of the 3-GMPM panel (AUC = 0.865 ± 0.101) versus the full set of 27 differential metabolites (AUC = 0.855 ± 0.095). **(D)** Correlation between the SVM-derived panel score and MMSE scores (Spearman r = −0.544, P < 0.001). **(E)** Incremental diagnostic performance evaluated by comparing logistic regression models with different feature sets: metabolite panel alone (AUC = 0.891), panel with age and sex (AUC = 0.891), and panel integrated with MMSE (AUC = 0.978). AUC, area under the curve; LASSO, least absolute shrinkage and selection operator; MMSE, Mini-Mental State Examination; ROC, receiver operating characteristic; SVM, support vector machine.

To identify a compact and robust diagnostic metabolite panel, LASSO logistic regression with stability selection was performed on the 10 GMPM candidates. The selection procedure was run for 200 iterations with 5-fold cross-validation, and metabolites with a selection frequency above 70% were retained for the final panel. Three metabolites—citric acid, 3-hydroxyoctanoic acid, and glyoxylic acid—were consistently selected across iterations, with selection frequencies of 78.4%, 73.5%, and 73.2%, respectively (Figure 5A). All three metabolites were involved in the TCA cycle and glyoxylate/dicarboxylate metabolism pathways, consistent with the core metabolic disturbances identified in the enrichment analysis. The abundance differences of the three panel metabolites between the AD and control groups were presented as box plots (Figure 5B).

The diagnostic performance of the 3-GMPM panel was evaluated using a support vector machine (SVM) classifier with repeated 5-fold cross-validation (10 repeats). The 3-metabolite panel achieved an AUC of 0.865 ± 0.101, which was slightly higher than that of the full set of 27 differential metabolites (AUC = 0.855 ± 0.095) (Figure 5C). To assess whether the panel’s discriminative performance was confounded by demographic imbalance between groups, we re-evaluated the SVM classifier with age and sex included as additional input features alongside the three panel metabolites. This demographic-adjusted model yielded an AUC of 0.852 (Supplementary Figure S1), which was minimally reduced relative to the metabolite-only model and did not improve discriminative performance. These results indicate that the diagnostic information captured by the three panel metabolites is largely independent of age and sex, supporting the robustness of the GMPM panel against demographic confounding. At the optimal classification threshold, the panel exhibited a sensitivity of 62.2%, a specificity of 100.0%, and an overall diagnostic accuracy of 83.5%. These findings demonstrate that feature selection guided by gut microbiota–metabolite association criteria effectively eliminate redundant noise and preserves core discriminative information, resulting in a compact diagnostic panel with comparable or moderately improved di-agnostic performance relative to the full set of differential metabolites. However, the large gap between specificity and sensitivity has clear clinical implications. Specifically, this asymmetry suggests the 3-GMPM panel is not suitable as a standalone diagnostic test, but rather should be used alongside established cognitive assessments (e.g., MMSE) and core biomarkers (e.g., pTau, Aβ42/40 ratio) within a tiered diagnostic workflow to support clinical decisions and reduce false negatives.

Spearman’s rank correlation was performed to assess the relationship between the SVM-derived panel score and clinical variables. For all participants with complete clinical data, the panel score showed a significant negative correlation with the MMSE score (r = −0.544, P < 0.001), indicating that higher panel scores were associated with more severe cognitive impairment (Figure 5D). In the AD group, the panel score exhibited a positive correlation with the CSF Aβ42/40 ratio that did not reach statistical significance (r = +0.381, P = 0.107, n = 25). This lack of statistical significance likely reflects the insufficient statistical power afforded by the small CSF biomarker subset (n = 25), precluding a definitive assessment of the relationship between the metabolic panel and core AD pathological markers.

The incremental diagnostic value of combining metabolomic and clinical variables was evaluated by comparing logistic regression models with different feature sets (Figure 5E). The metabolite panel alone yielded a cross-validation AUC of 0.891. The addition of age and sex did not improve discriminative performance, suggesting that the metabolite panel captured most of the diagnostic information. The integrated model, combining the panel score with MMSE, performed substantially better (AUC = 0.978), indicating concordance between the metabolite panel and clinical cognitive assessment.

## 4 Discussion

In this study, we constructed a three GMPM panel using four complementary UPLC-MS/MS plasma metabolomics platforms in conjunction with fecal metagenomic sequencing. Compared with previous plasma metabolite panels, our GMPM panel demonstrated comparable discriminative performance (AUC = 0.865, specificity 100.0%), positioning it as a confirmatory auxiliary tool for distinguishing AD from controls. The metabolites included in our GMPM panel are characteristic molecules that are likely co-altered by gut microbiota and AD pathophysiology. The etiology of AD remains unclear and is influenced by multiple factors, including aging, environmental exposures, and genetic predisposition (Breijyeh and Karaman, 2020; Chen and Holtzman, 2022; Sierksma et al., 2020). Emerging evidence suggests that dysregulation of gut microbial abundance, diversity, and function may serve as a critical factor in AD pathogenesis through modulation of cognition-related neural circuits (Zhao H. et al., 2025; Varesi et al., 2022; Leblhuber et al., 2021; Megur et al., 2020).

Previous studies have reported differences in gut microbiota between AD patients and control subjects across diverse populations (Vogt et al., 2017; Zhuang et al., 2018; Kowalski and Mulak, 2019). These findings support the hypothesis that the gut-brain axis enables bidirectional communication between gut microbiota and the central nervous system (CNS) through neural, endocrine, and immune pathways (Fung et al., 2017; Cryan and Dinan, 2012). In the present study, we observed whereas neither α-diversity nor β-diversity showed significant differences between groups. Although some studies have reported reduced gut microbial diversity in AD patients (Hung et al., 2022), our findings are consistent with previous reports (Sepulveda-Rivera et al., 2025), suggesting that gut microbiota dysbiosis in AD may manifest as subtle and specific taxonomic changes rather than a global collapse or drastic restructuring of the entire community. Most notably, we found a nominal decrease in *F. duncaniae* and a nominal enrichment of pro-inflammatory taxa, including *C. leptum* and *R. bovis*, which is directionally consistent with prior studies (Sepulveda-Rivera et al., 2025; Cattaneo et al., 2017). However, it is critical to note that none of these species remained statistically significant after FDR correction, indicating that the magnitude of microbiota alterations in our cohort was modest relative to the substantial inter-individual variation inherent to gut microbial communities. The genus *Faecalibacterium* represents one of the most abundant butyrate-producing bacteria in the human colon. Butyrate has been reported to play crucial roles in intestinal physiology through its anti-inflammatory properties, enhancement of intestinal barrier function, and promotion of mucosal immunity. In our cohort, *F. duncaniae* exhibited the strongest LEfSe discriminatory capacity (LDA = 3.22) and was the most pronounced depleted species (log_2_ fold-change = −0.878, uncorrected P = 0.036). While this finding did not survive FDR correction, its directional consistency with established literature—where reduced *Faecalibacterium* abundance has been associated with compromised intestinal barrier integrity, exacerbated systemic inflammation, enhanced neuroinflammation, and accelerated AD progression (Liu et al., 2018)—suggests that it represents a biologically plausible candidate warranting validation in larger, adequately powered cohorts. Correlation analysis further revealed that multiple GMPMs were significantly correlated with *Limosilactobacillus fermentum*, *S. rubneri*, and *F. duncaniae*. *Limosilactobacillus fermentum* and *S. rubneri*, as beneficial bacteria, have been reported to contribute to strengthening intestinal barrier function, reducing abnormal accumulation of metabolites such as uric acid and blood ammonia, and activating immune responses (Ozen et al., 2023; Liu et al., 2021). Correlation analysis further revealed that multiple GMPMs were correlated with *F. duncaniae*, *Limosilactobacillus fermentum*, and *S. rubneri* at an uncorrected threshold. Collectively, the microbiome findings of this study are best characterized as hypothesis-generating rather than confirmatory. The high inter-individual heterogeneity of gut microbial communities, combined with the modest sample size (n = 72 for the multi-omics subset), likely constrained statistical power to detect reproducible species-level differences. Future studies incorporating larger cohorts, longitudinal sampling, and functional validation (e.g., metatranscriptomics or gnotobiotic models) will be essential to establish potential causal links between specific gut microbial taxa and peripheral metabolic alterations in AD. These results provide a basis for hypothesizing that gut-associated plasma metabolites may be involved in AD pathogenesis and support the exploratory potential of blood-based, microbiota-related metabolites as candidate diagnostic biomarkers for AD.

The three metabolites constituting our diagnostic panel—citric acid, 3-hydroxyoctanoic acid, and glyoxylic acid—are closely associated with TCA cycle and glyoxylate shunt (Kalecky et al., 2022; Cronan and Laporte, 2005). This association is suggestive of a link to the characteristic mitochondrial dysfunction and impaired cerebral energy metabolism reported in AD pathogenesis (Sang et al., 2022). Citric acid, the first intermediate of the TCA cycle, is formed by the condensation of acetyl-coenzyme A (acetyl-CoA) and oxaloacetate, serving as a key metabolic node that bridges the oxidative catabolism of major nutrients and multiple biosynthetic pathways. The decreased plasma citric acid levels observed in AD patients in our cohort are consistent with previous studies (Yao et al., 2009; Khan et al., 2000). Peripheral depletion of citric acid may potentially reflect a systemic shift of the organism from classical oxidative phosphorylation toward less efficient alternative adenosine triphosphate (ATP) generation pathways. Notably, prior investigations have demonstrated that cellular senescence in the brain and abnormal glucose metabolism represent early pathological features of AD, potentially preceding classical pathological changes such as Aβ deposition and tau hyperphosphorylation (Wang et al., 2022; Butterfield and Halliwell, 2019; Huo et al., 2020). Therefore, alterations in plasma citric acid levels may serve as a candidate indicator for early metabolic reprogramming in AD. Glyoxylic acid is a highly reactive aldehyde that can be generated through multiple processes, including glycolate oxidation and amino acid catabolism. Dysregulation of glyoxylate and dicarboxylate metabolism has been hypothesized to potentially lead to excessive production of reactive oxygen species (ROS), thereby contributing to oxidative stress (Di Meo et al., 2016). The role of glyoxylate/dicarboxylate metabolism in oxidative stress regulation has been implicated in substantial evidence implicating oxidative stress participates in AD pathophysiology (Huang et al., 2016; Dhapola et al., 2024; Wang et al., 2014). Clinical and experimental studies have demonstrated that increased oxidative stress and altered energy metabolism are associated with amyloid-β aggregation, tau hyperphosphorylation, and synaptic dysfunction—all hallmark features of AD (Dhapola et al., 2024; Butterfield et al., 2001; Swerdlow, 2018). In our study, the concurrent depletion of glyoxylic acid and citric acid in AD patients is consistent with the interpretation of an overall reduction in the activity of the TCA cycle and glyoxylate/dicarboxylate metabolic pathways. This metabolic pattern is aligned with the hypothesis that mitochondrial dysfunction leads to excessive ROS production and oxidative stress during AD pathological progression, subsequently exacerbating Aβ synthesis and tau hyperphosphorylation (Yao et al., 2009; Resende et al., 2008; Egana et al., 2003; Gomez-Ramos et al., 2003). 3-Hydroxyoctanoic acid is a medium-chain fatty acid that can activate G protein-coupled receptor 84 (GPR84), thereby promoting chemotaxis of leukocytes and macrophages and the release of pro-inflammatory cytokines (Pete et al., 2022; Peters et al., 2020). Our study found significantly elevated plasma 3-hydroxyoctanoic acid levels in AD patients, which is suggestive of a potential involvement of this metabolite may be involved in the regulation of AD-associated peripheral or central inflammatory microenvironments. It is important to emphasize that these mechanistic inferences are derived from observational associations in the present study and rely on evidence from previously published experimental literature. Direct functional validation—such as cell-based assays or animal models—is required to establish causal relationships between these metabolic alterations and AD pathophysiology.

The field of blood-based biomarkers for AD has developed rapidly in recent years. pTau181, pTau217, plasma Aβ42/40 ratio, and NFL have gradually emerged as minimally invasive alternatives that demonstrate high concordance with CSF and PET molecular imaging results (J et al., 2020; Nakamura et al., 2018; Palmqvist et al., 2020; Lan et al., 2025; Smirnov et al., 2022). Additionally, established plasma biomarkers—including the emerging astrocytic marker GFAP—primarily reflect central proteinopathy and neuronal injury, including Aβ plaque deposition, pathological tau tangles, and neuronal axonal injury, whereas the 3-GMPM panel captures peripheral energy metabolism and gut microbial function, representing a distinct, complementary diagnostic dimension. In this study, by screening metabolites specifically associated with AD-characteristic gut microbiota alterations, we constructed a diagnostic panel comprising three gut microbiota-related plasma metabolites, achieving a cross-validated AUC of 0.865—a value superior to that of the model constructed using all 27 differential plasma metabolites. For example, a recent study constructed a predictive model incorporating eight plasma metabolites, achieving an AUC of approximately 0.861 for discriminating AD from controls (Qiang et al., 2024). Another targeted metabolomics study reported a cross-validated AUC of 0.82 using plasma signature metabolites combined with basic sociodemographic data (sex, age, education, BMI, and APOE ε4 status) (Kalecky et al., 2022). A study based on the AD Neuroimaging Initiative (ADNI) cohort utilized UPLC-MS/MS metabolomics and deep learning approaches, wherein 21 metabolic markers selected by the LASSO algorithm yielded a neural network model with an AUC of 0.873 (Wang et al., 2023). Furthermore, when the 3-GMPM MetRS was integrated with MMSE scores in a logistic regression model, the AUC increased significantly to 0.978. However, this figure should be interpreted cautiously given the strong collinearity between MMSE and diagnostic group assignment, and it primarily illustrates concordance between peripheral metabolic signatures and clinical cognitive assessment rather than independent incremental diagnostic value of the panel. It is also notable that the 3-GMPM panel score showed no statistically significant correlation with the CSF Aβ42/40 ratio in the AD subgroup (r = +0.381, P = 0.107, n = 25). This null result precludes a definitive conclusion regarding the relationship between the gut-microbiota-associated metabolic panel and core AD pathological markers, underscoring that the 3-GMPM panel captures peripheral metabolic dysregulation that is not directly aligned with central amyloid pathology as measured by CSF biomarkers. This divergence is consistent with the distinct biological dimensions targeted by our panel (peripheral energy metabolism and gut microbial function) versus established biomarkers (central Aβ and tau pathology). These results demonstrate that focusing on microbiota-associated metabolic signatures can effectively eliminate redundant noise while preserving core discriminative information, thereby validating the rationale of our strategy to explore AD diagnostic biomarkers from the perspective of the gut-brain metabolic axis.

Given its high specificity (100.0%) but low sensitivity (62.2%), the 3-GMPM panel is better suited as a “rule-in” tool for confirming AD in suspected cases—where false positives are minimised—rather than for broad community-based screening, where its low sensitivity would limit its utility. The panel correctly classified all 48 control participants, thereby reducing unnecessary cerebrospinal fluid examinations or costly PET scans in specialist settings. However, the modest sensitivity implies that more than one-third of real AD patients would be misclassified as non-diseased by the panel alone, carrying substantial clinical risk. Consequently, the 3-GMPM panel should not be used as a standalone diagnostic criterion for AD; its appropriate role lies as an adjunctive confirmatory modality integrated with established cognitive assessments (e.g., MMSE) and core biomarkers (e.g., pTau, Aβ42/40 ratio). The current findings were derived from a single-center cohort with modest sample size (n = 85), and the repeated 5-fold cross-validation framework, while robust against overfitting, does not substitute for true external validation. Several priority directions for future research should be emphasized. First, large-scale, multi-center prospective studies encompassing diverse ethnicities, age strata, and comorbidity profiles are essential to establish the generalizability and stability of the identified metabolite thresholds. Second, the 3-GMPM panel must be validated in individuals with mild cognitive impairment (MCI)—the prodromal stage of AD—to determine whether the gut-microbiota–metabolite axis is perturbed sufficiently early to support preclinical screening and risk stratification. Given that MCI represents a heterogeneous population with variable conversion rates to dementia, longitudinal cohort designs with serial metabolomic and microbiomic sampling will be required to assess the panel’s predictive value for disease progression. Third, future investigations should integrate the 3-GMPM panel with emerging blood-based biomarkers (e.g., pTau217) and neuroimaging modalities to construct a composite, multi-domain risk algorithm aligned with the AT(N) framework. Finally, mechanistic studies employing *in vitro* and gnotobiotic models are needed to establish whether the observed metabolic shifts are causally linked to AD pathophysiology or represent secondary epiphenomena, thereby informing the development of microbiota-targeted therapeutic strategies.

However, several limitations of the current study should be acknowledged. First, the sample size is relatively small, particularly for the multi-omics subset with paired fecal and plasma samples (n = 72). Although stringent stability selection and repeated cross-validation were employed to mitigate overfitting to some extent, the generalizability of the 3-GMPM diagnostic panel remains to be independently validated in larger, multi-center cohorts with diverse ethnic and demographic backgrounds. The AD group also exhibited a significant sex imbalance compared with controls. While diagnostic models adjusted for age and sex showed comparable performance, this demographic discrepancy and the lack of data on dietary patterns, gastrointestinal medications, diabetes, hypertension, and APOE genotype remain unaddressed confounders. Second, the cross-sectional design of this study precludes inferences about the dynamic interplay between metabolism and the microbiome. Consequently, we cannot determine whether the observed plasma metabolite alterations represent pathophysiological changes that precede cognitive impairment or are merely secondary consequences of established AD pathology. Nor can we assess their utility in predicting future cognitive decline or conversion to MCI or AD. Third, the 3-GMPM panel score showed no significant correlation with MMSE scores within the pure AD patient subgroup, indicating that it is more appropriate for distinguishing AD from healthy controls rather than evaluating the severity of cognitive impairment. Fourth, the cerebrospinal fluid Aβ42/40 subgroup included fewer than 30 participants, providing limited statistical power to fully elucidate the relationship between the metabolomic panel and core AD pathological markers. Finally, the diagnostic sensitivity of the panel warrants further improvement. Overall, the 3-GMPM panel developed in this study is more appropriately positioned as an auxiliary diagnostic tool for AD rather than a standalone diagnostic indicator, and its clinical utility requires further validation in larger multi-center cohorts. To address the inherent limitations of our cross-sectional design, prospective longitudinal cohort studies—incorporating serial biosampling at preclinical, MCI, and dementia stages—should be prioritized as a critical next step to determine whether microbiome-associated metabolic signatures exhibit temporal dynamics predictive of cognitive trajectory and diagnostic conversion.

## 5 Conclusion

In this study, we integrated targeted plasma metabolomics with fecal metagenomic sequencing to identify a three-metabolite diagnostic panel (citric acid, 3-hydroxyoctanoic acid, and glyoxylic acid) for Alzheimer’s disease. The panel demonstrated a cross-validated AUC of 0.865, with high specificity (100.0%) but modest sensitivity (62.2%), positioning it as a confirmatory auxiliary tool for distinguishing AD from cognitively normal controls rather than a standalone diagnostic standard or broad screening instrument. Notably, the observed specificity was derived from a single-center cohort with modest sample size and is likely to decrease in real-world, multi-center settings. Our findings underscore the critical role of the gut–brain metabolic axis in AD and highlight the complementary value of microbiota-linked peripheral metabolic signatures relative to established central-pathology biomarkers (e.g., pTau, Aβ42/40 radio). However, the study was limited by a significant sex imbalance between groups, a cross-sectional design, and the absence of external validation. Future prospective studies in larger, multi-center, and ethnically diverse cohorts—including individuals with mild cognitive impairment (MCI)—are warranted to validate the panel’s generalizability, assess its utility for early-stage detection, and examine its performance alongside emerging blood-based biomarkers within a multi-dimensional diagnostic framework.

## Data Availability

Detailed repository information, including the Zenodo DOI and raw data availability, is provided in the Supplementary Material.
