# Supplementary material for: Multi-omics integration identifies a gut-microbiota-associated plasma metabolite signature for alzheimer disease diagnosis and metabolic pathway targeting
Source: Front Pharmacol. 2026 Aug 19;17:1933454. doi: 10.3389/fphar.2026.1933454 (PMC13533937; doi:10.3389/fphar.2026.1933454)
Supplement: Supplementary file 1 [file Supplementaryfile1.docx]

Supplementary Material

**Table S1.** Cross-tabulation of the 3-GMPM panel classification against the clinical diagnosis.

|  | **AD (n = 37)** | **Control (n = 48)** |
| --- | --- | --- |
| Panel positive | 23 | 0 |
| Panel negative | 14 | 48 |

Note. Sensitivity: 62.2%; Specificity: 100.0%; Positive predictive value: 100.0%;

Negative predictive value: 77.4%; Overall accuracy: 83.5%.

GMPM = gut-microbiota-associated plasma metabolite; AD = Alzheimer's disease.

**Figure S1. Diagnostic performance of the 3-GMPM panel with and without demographic covariates.** Receiver operating characteristic (ROC) curves comparing the 3-GMPM metabolite panel (AUC = 0.864) versus the 3-GMPM panel with age and sex included as additional covariates (AUC = 0.852), evaluated using a support vector machine (SVM) classifier with repeated 5-fold cross-validation (10 repeats). AUC, area under the curve.

**Data Availability**

The processed datasets analyzed in this study, including the plasma metabolite abundance matrix, fecal metagenomic OTU table, and diversity analysis results, are available in the Zenodo repository (DOI: 10.5281/zenodo.21284447) upon publication. Raw metabolomics data (LC-MS/MS raw files) and raw metagenomic sequencing data (FASTQ files) are available from the corresponding author upon reasonable request due to file size limitations. Clinical data are available from the corresponding author upon reasonable request and IRB approval.
